# Primary care-based post diagnostic dementia care in England: modelling and scaling up evidence-based models for efficient future care

**DOI:** 10.1093/ageing/afag232

**Published:** 2026-08-10

**Authors:** Magdalena Walbaum, Derek King, Louise Robinson, Martin Knapp, Raphael Wittenberg

**Affiliations:** Care Policy and Evaluation Centre - The London School of Economics and Political Science, London, United Kingdom; Care Policy and Evaluation Centre - The London School of Economics and Political Science, London, United Kingdom; Population Health Sciences - Newcastle University, Newcastle upon Tyne, United Kingdom; Care Policy and Evaluation Centre - The London School of Economics and Political Science, London, United Kingdom; Care Policy and Evaluation Centre - The London School of Economics and Political Science, London, United Kingdom

**Keywords:** dementia, post-diagnostic support, health economics, older people

## Abstract

**Background:**

The global prevalence of dementia is rising rapidly, placing increasing pressure on health and social care systems to deliver coordinated and equitable post-diagnostic support. In England, service fragmentation and workforce constraints limit the delivery of recommended care models. Non-clinical dementia navigator (DN) models have emerged as a potentially scalable approach to improve care coordination and access, yet evidence on their system-level impact remains limited.

**Methods:**

We estimated the population of people with diagnosed dementia in England eligible for DN support between 2025 and 2030 using NHS data and projected prevalence trends. Eligibility was adjusted for undiagnosed cases, care home residents, and expected uptake. Costs of delivering DN-led care were derived from existing UK service models and unit costs, while potential savings were based on reductions in health-care utilisation observed in the US Care Ecosystem trial. Probabilistic sensitivity analyses were conducted to assess uncertainty.

**Results:**

The number of eligible individuals receiving DN support is projected to increase from 300 000 in 2025 to 360 000 in 2030. Delivering this model would require ~940 to 1120 link workers. Annual costs are estimated at £224 million in 2025, rising to £343 million in 2030. These are offset by estimated savings of £1140 million and £1750 million, respectively. Even under conservative assumptions, DN models generate net savings.

**Conclusions:**

Scaling up DN-led care in England could support large numbers of people with dementia while generating substantial health system savings. These findings suggest that navigator-based models offer a feasible and cost-saving approach to delivering coordinated post-diagnostic dementia care at scale.

## Key Points

- Dementia navigator models could support >300 000 people within primary and community care systems.
- Navigator-led care reduces health-care utilisation and generates net cost savings under conservative assumptions.
- These models improve care coordination and address workforce constraints in post-diagnostic dementia care.

## Introduction

Global prevalence of dementia is rising rapidly [1], placing increasing pressure on health and social care systems to deliver timely, coordinated, equitable support [2]. While recent advances in research in disease-modifying treatments for Alzheimer’s disease generate optimism, few people with dementia are currently eligible for them [3]. For most people, quality of life and health outcomes depend on availability and effectiveness of post-diagnostic care and ongoing support [3]. Yet evidence from countries like England consistently highlights geographical variation, service fragmentation, and limited continuity of care, with particularly pronounced gaps for people from socio-economically disadvantaged or ethnic minority communities, and those living in rural/remote areas [2, 4, 5].

Evidence-based clinical guidance is limited in recommending care models [6, 7]. Guidance in England recommends that people with dementia should have access to a named clinical professional to coordinate care from diagnosis to end of life, supported by personalised care planning and regular review [6]. However, delivering this model at scale using exclusively clinical staff presents significant workforce and budgetary challenges [3]. Non-clinical dementia navigators—also referred to as dementia advisers or link workers—have emerged as a potentially scalable solution to improve care coordination, continuity, and access to support [8].

Dementia navigator (DN) models typically provide a single, consistent point of contact for people with dementia and their unpaid carers, offering information, emotional support, needs assessment, and proactive signposting to local services, but there is limited evidence on their effectiveness [8, 9]. At present such roles exist in England in varied and fragmented forms such as dementia advisers, link workers and social prescribers in both clinical and research settings [10], but are unevenly implemented and not universally available [8, 11]. A systematic review of dementia care navigation found that, although promising, the evidence base is mixed and methodologically heterogeneous, with the US Care Ecosystem the only randomised controlled trial identified [8]. Evidence from the US-based Care Ecosystem model [12–14], suggests that navigator-led approaches embedded within multidisciplinary teams can improve quality of life for people with dementia, reduce carer burden, and lower avoidable health-care utilisation. These benefits were achieved alongside net cost savings, particularly among people with greater clinical and social complexity [13, 14].

We explored the scalability of DN models in England, which has primary care as first point of patient contact, by estimating the population eligible to receive navigator-led support, the workforce required to deliver it, and the associated economic implications over the medium term.

## Methods

We estimated the number of people with diagnosed dementia in England eligible to receive DN support between 2025 and 2030. Baseline prevalence in 2025 was derived from National Health Service (NHS) data [15]. To project future prevalence, we assumed that the average annual increase in number of people with dementia between 2022 and 2025 would continue linearly to 2030. We then made three sequential subtractions to estimate the population eligible for dementia navigator support. We excluded undiagnosed cases by applying the recorded NHS diagnosis rate of 66.5%, thereby removing the estimated 33.5% of people with dementia who are undiagnosed [15]; individuals living in care homes (34.2% of people with dementia) [15], and 8% of the remainder who we assumed would decline participation, based on the Care Ecosystem trial [13]. Eligibility for the modelled DN support was therefore defined as: recorded dementia diagnosis, not resident in a care home, and not declining support.

To estimate the cost of scaling-up this model of care we evaluated multiple existing primary care-led and community-based models of post-diagnostic support delivered by DNs, social prescribers, dementia advisers, and clinical leads [11], including established primary care navigator programmes in England employing non-clinical staff studied through Primary care-led post diagnostic Dementia Care (PriDEM) [16], as well as the Care Ecosystem approach [13, 14, 17]. We identified and reconciled staff roles involved in delivering navigator-led care, across the different models of support, assessing all staffing components required for delivery. Time inputs for delivery, supervision, training, and coordination activities were estimated through interviews and correspondence with practices that had introduced these support models. Support costs were derived by multiplying time inputs by UK unit costs for health and social care staff roles [18]. We modelled a primary care-based navigator team rather than a single mental health Trust, consistent with primary care being the first point of contact in England. Costs were taken from the Personal Social Services Research Unit (PSSRU) Unit Costs of Health and Social Care [16]; these are fully absorbed unit costs that include salary on-costs, qualifications, and capital, accommodation and administrative/management overheads. Accommodation and infrastructure support are therefore captured within the per-person costs rather than excluded.

We calculated average per-person annual cost of DN support across the different models, reflecting variation in skill mix and service configuration. This approach was intended to produce a pragmatic estimate suitable for national scale-up rather than modelling a single service configuration. Costs were expressed in Great British Pounds (GBP), inflated to 2025 prices [19]. Salary costs were uprated using explicit assumptions on recent and expected NHS pay settlements, implying real-terms growth in staff costs over time (Table 1).

Potential savings associated with DN support were based on findings from the Care Ecosystem trial, which reported reductions in overall health-care costs among people with dementia receiving navigator-led, team-based care. We applied an annual per-person saving of £4106 (95% confidence interval (CI) £350 to £7831, sample size 460 dyads at 2025 prices), derived from mean cost reduction reported in the US trial and converted from USD to GBP using purchasing power parity exchange rate as of 2024 (0.66 GBP per international dollar). Savings were assumed to accrue from reduced emergency department use, hospital admissions, and other health-care utilisation.

Probabilistic sensitivity analyses tested for robustness of results to changes in key parameters. Given uncertainty in the rate at which eligible people with dementia decline DN support, we varied this parameter by ±20% assuming a normal distribution. Costs were varied by ±20% around base-case values using a gamma distribution, in line with standard practice [20]. We also modelled best- and worst-case scenarios for savings by applying upper and lower bounds of the 95% CI reported in the Care Ecosystem evaluation. Analyses were conducted in Microsoft Excel (Office 365), using 1000 Monte Carlo simulations, with results summarised as means and 95% uncertainty intervals defined by 2.5th and 97.5th percentiles of simulated distributions. Parameters, base-case values, distributions, ranges and sources used in the costing and probabilistic analysis are summarised in Appendix 1 of Supplementary material.

**Table 1:** Cost of intervention.

Staff	Annual salary^a^	Per person^**b**^
Cost link worker	£37 160	£186
Costs supervisor	£79 524	£20
Full time social worker	£85 174	£426
Full time nurse	£79 524	£398
Part time pharmacist	£83 039	£208

^a^Annual salary assuming a fulltime staff.

^b^Assuming a workload of 1 link worker per 323 persons with dementia based on calculations from data provided by six English local sites

## Results

The number of older people with dementia in England is projected to increase from ~750 000 in 2025 to 900 000 in 2030 (Table 2). Accounting for people with dementia not diagnosed and declining or ineligible for DN support, the estimated number of people with dementia receiving DN care would rise from 300 000 in 2025 to 360 000 in 2030.

**Table 2:** Number of people with dementia receiving DN care, costs and savings.

Parameters	2025	2026	2027	2028	2029	2030
Annual overall people living with dementia	749 400	777 182	805 994	835 874	866 862	898 998
Estimated annual number using DN care	302 239	313 574	325 149	337 135	349 671	361 905
Estimated number of link workers	936	971	1007	1044	1083	1120
Estimated cost of model of care per person	£804 (£792 - £817)	£845 (£832—£856)	£886 (£874—£899)	£931 (£918—£945)	£978 (£963—£992)	£1027 (£1012—£1041)
Annual total cost^a^	£224 (£220—£228)	£244 (£240—£248)	£266 (£262—£270)	£289 (£285—£294)	£315 (£310—£320)	£343 (£337—£348)
Annual savings base case^a^	£1139 (£1047—£1216)	£1248 (£1164—£1329)	£1359 (£1264—£1452)	£1470 (£1366—£1561)	£1613 (£1501—£1722)	£1750 (£1629—£1862)
Annual savings worst case^a^	£106 (£98—£113)	£116 (£108—£123)	£125 (£116—£134)	£137 (£127—£146)	£149 (£138—£160)	£163 (£151 - £173)
Annual savings best case^a^	£2167 (£2015—£2310)	£2384 (£2199—£2557)	£2589 (£2402—£2760)	£2816 (£2613—£3003)	£3089 (£2877—£3300)	£3331 (£3093—£3572)

^a^Annual costs and savings of model of care are presented in millions £ at constant 2025 prices. Results are reported as means with 95% uncertainty intervals derived from the 2.5th and 97.5th percentiles of the PSA

To deliver the intervention to eligible people, 940 link workers would be needed in 2025 and 1120 in 2030, assuming workload of 323 people with dementia per link worker annually (based on calculations from data provided by six English local sites).

Provision of DN care to eligible people is estimated to cost £224 million in 2025, generating total annual savings of £1140 million. By 2030, annual DN care costs would increase to £343 million, with total annual savings rising to £1750 million.

Scenario analyses demonstrated substantial uncertainty around the magnitude of potential savings, driven primarily by variation in service-use reductions. Under a conservative (worst-case) scenario, applying the lower bound of the 95% CI for savings in the Care Ecosystem trial, DN models generated net savings of £106 million in 2025, increasing to £163 million by 2030. In contrast, under a best-case scenario based on the upper bound of the trial’s CI, estimated net savings were substantially higher (£2170 million in 2025, and £3330 million by 2030).

## Discussion

This study provides system-level evidence on the potential savings of scaling up primary care-based, DN models in England, suggesting that navigator-led post-diagnostic support could be delivered at scale while generating net savings to the health system. This work is timely, given the new national policy incorporating a dementia-specific service framework [21]. We estimate that DN models could have supported > 300 000 people with dementia in 2025, rising to >360 000 by 2030. Under base-case assumptions, costs of provision are more than offset by reductions in health-care utilisation, with scenario analyses indicating that DN models would be cost saving even under conservative assumptions.

Because UK effectiveness is not yet established, with the only relevant research being a mixed methods implementations study [10], these estimates should be read as conditional and exploratory, indicating the potential economic impact if the effectiveness observed in the Care Ecosystem trial transfers to England, rather than as a basis for immediate scale-up. As highlighted by Giebel et al [8], the evidence base for navigator models remains mixed, and our savings estimates rest on a single randomised controlled trial. The reliance on US trial data strengthens, rather than weakens, the case for a UK effectiveness and implementation trial before, or alongside, wider implementation. Beyond cost, implementation will raise challenges including workforce recruitment and retention, training and supervision capacity, integration with existing services, and fidelity.

Several limitations should be acknowledged. Estimated savings are derived from a single US trial and adjusted to the UK context. Care Ecosystem eligibility required enrolment in, or eligibility for, Medicare or Medicaid rather than private insurance, and cost outcomes were assessed among Medicare beneficiaries [11, 12]; the sample was therefore not restricted to the privately insured. Because participants were not charged for the service, the assumed decline rate is unlikely to reflect cost or ability to pay, and reported attrition was driven predominantly by death rather than financial factors. Nonetheless, differences in health-system structure and the absence of conclusive UK effectiveness evidence mean caution is warranted in attributing all projected savings to the model. While sensitivity analyses explored uncertainty, realised savings in England may differ depending on local service configuration, fidelity of implementation, and integration with existing services. Second, our analyses focused on health-care costs and do not capture impacts on social care, unpaid carers or other budgets, which are likely to be substantial and unevenly distributed across population groups. Third, we modelled average per-person cost across heterogeneous DN configurations; actual costs and impacts will vary by skill mix, intensity of support and local need.

Workforce requirements identified here suggest that DN models are potentially scalable within existing primary and community care infrastructures, particularly if aligned with wider developments in social prescribing and integrated care, as care is shifted more into the community [21]. Scaling-up would require workforce expansion, additional training capacity and potentially higher remuneration to recruit and retain staff. While we assume that demand for post-diagnostic support will rise over time in line with demographic trends, demand may increase more substantially if DN services are perceived as more accessible and responsive than existing provision.

Our findings align with international evidence that navigator-led, team-based approaches can improve care coordination and continuity, reduce avoidable hospital emergency attendances and admissions, and alleviate carer burden, particularly for people with greater clinical and social complexity [13, 14]. The scale of estimated savings reflects the ability of non-clinical roles to undertake navigation, coordination and proactive support functions that otherwise fall to higher-cost clinical staff as currently recommended [6]. This is especially pertinent given rising dementia prevalence, constrained NHS capacity and persistent workforce shortages [21].

From a policy perspective, DN models offer a pragmatic route to operationalising national ambitions for high-quality post-diagnostic dementia care. DN models, embedded within multidisciplinary systems and with clear routes for escalation to clinical care, may help reconcile quality aspirations with workforce and budgetary constraints [8, 9, 13].

## Supplementary Material

aa-26-0860-File002_afag232
