# Supplementary material for: Primary care-based post diagnostic dementia care in England: modelling and scaling up evidence-based models for efficient future care
Source: Age Ageing. 2026 Aug 10;55(8):afag232. doi: 10.1093/ageing/afag232 (PMC13456331; doi:10.1093/ageing/afag232)
Supplement: Supplementary file 1 [file aa-26-0860-file002_afag232.docx]

**Primary care-based post diagnostic dementia care in England: modelling and scaling up evidence-based models for efficient future care**

Supplementary material

Appendix1: Parameters, distributions, ranges and sources used in the costing and probabilistic sensitivity analysis

| **Parameters** | **Base-case value** | **Distribution** | **Range** | **Source** |
| --- | --- | --- | --- | --- |
| Care home residents (excluded) | 34.2% | n/a | n/a | NHS data [14] |
| Undiagnosed (excluded) | 33.5% | n/a | n/a | NHS data [14] |
| Decline participation | 8% | Normal | ±20% | Possin et al [12] |
| Per-person annual saving | £4,106 | n/a | 95% CI £350 to £7,831 | Guterman et al [13] |
| Workload per link worker | 323 person/year* | n/a | n/a | Six English sites |
| Staff/unit costs (see Table 1) | n/a | Gamma | ±20% | PSSRU; Jones et al [16] |
| PPP conversion (USD to GBP) | 0.66 | n/a | n/a | 2024 PPP rate |

*Person with dementia
